# Cord Blood Serum (CBS)-Based Eye Drops Modulate Light-Induced Neurodegeneration in Albino Rat Retinas

**DOI:** 10.3390/biom10050678

**Published:** 2020-04-28

**Authors:** Stefano Di Marco, Serena Riccitelli, Mattia Di Paolo, Emilio Campos, Marina Buzzi, Silvia Bisti, Piera Versura

**Affiliations:** 1 Department of Applied Clinical Science and Biotechnology, University of L’Aquila, Via Vetoio, Coppito II, 67100 L’Aquila, Italy; 2 Istituto Nazionale di Biostrutture e Biosistemi (INBB), Via Medaglie d’Oro 305, 00136 Roma, Italy; 3 Center for Synaptic Neuroscience and Technology, Istituto Italiano di Tecnologia, Largo Rosanna Benzi, 16132 Genova, Italy; 4 IRCCS, Ospedale Policlinico San Martino, Largo Rosanna Benzi, 16132 Genova, Italy; 5 Ophthalmology Unit, University of Bologna and S. Orsola-Malpighi Teaching Hospital, 40138 Bologna, Italy; 6 Emilia Romagna, Cord Blood Bank-Transfusion Service, S. Orsola-Malpighi Teaching Hospital, 40138 Bologna, Italy; 7 NetS3 Laboratory, Istituto Italiano di Tecnologia, Via Morego, 30, 16163 Genova, Italy

**Keywords:** photoreceptor neurodegeneration, retina, trophic factor, cord blood serum

## Abstract

Age-related macular degeneration (AMD) is one of the leading causes of visual loss in western countries, it has no cure, and its incidence will grow in the future, for the overall population aging. Albino rats with retinal degeneration induced by exposure to high-intensity light (light-damage, LD) have been extensively used as a model of AMD to test neuroprotective agents. Among them, trophic factors (NGF and BDNF) have been shown to play a significant role in photoreceptors’ survival. Interestingly, cord blood serum (CBS) is an extract full of chemokines and trophic factors; we, therefore, hypothesized that CBS could be an excellent candidate for neuroprotection. Here, we investigate whether CBS-based eye drops might mitigate the effects of light-induced retinal degeneration in albino rats. CBS treatment significantly preserved flash-electroretinogram (f-ERG) response after LD and reduced the “hot-spot” extension. Besides, CBS-treated animals better preserved the morphology of the outer nuclear layer, together with a reduction in microglia migration and activation. Interestingly, the treatment did not modulate reactive gliosis and activation of the self-protective mechanism (FGF2). In conclusion, our results suggest that CBS-based eye drops might be successfully used to mitigate retinal neurodegenerative processes such as AMD.

## 1. Introduction

A variety of factors, including gene mutations, environmental stress, dysmetabolic problems, aging, and a combination of all of them, might induce retinal neurodegenerative processes. The outcome is impairment and loss of visual function due to neuronal death (photoreceptors, ganglion cells), neuroinflammation, mitochondrial dysfunction, synaptic remodeling, and more [1]. No effective clinical treatments exist for most retinal degenerative disorders. It remains a challenge to select treatments, which might cope with satisfactory results in neurodegenerative processes. The first problem to solve is to choose an appropriate experimental model to test different compounds. One model widely used is the albino rat, where high light exposure induces retinal degeneration. This model has been validated in several experiments (see [2,3] for reference) and the time course of photoreceptor degeneration, morphological, and functional changes clearly described. The first event is phototoxic [4] and triggers a series of processes leading to photoreceptor death by apoptosis in a restricted dorsal area named the “hot spot”. The degeneration progresses in time and space following the activation of “down-stream events”, including glial reaction, cytokine release, neuroinflammation, etc. [5]. This model is reminiscent of the histopathology of age-related macular degeneration (AMD) [3]. In light-damage (LD) rats, many neuroprotectants have been tested from saffron [6] to crocetin [7], to cerium oxide nanoparticles [8] and neurotrophic factors like NGF [9] and BDNF [10]. Interestingly, cord blood serum is a balanced mixture of trophic factors and cytokines, and we hypothesize that cord blood serum (CBS) might be beneficial to ameliorate neurodegenerative processes. Recently, cord blood serum (CBS) based eye drops [11,12] have been used to repair cornea in severe epithelial damage, mainly looking at the high content of the epidermal growth factor (EGF). Other elements present in CBS, like NGF and BDNF, have already been separately tested [9,10]. Indeed, with CBS, we have the opportunity to evaluate a natural combination of factors. The possibility exists that according to differential contents of factors, we might select eye drops with targeted efficacy to different neurodegenerative processes and stages of the disease. Clinical observation in glaucoma patients [13] seems to support this hypothesis. Here we report preliminary data showing that treatment with CBS-based eye drops can ameliorate the retinal damage induced by exposure to high-intensity light.

## 2. Materials and Methods

### 2.1. Collection and Preparation of CBS Eye Drops

We used a complete quality system and excellent manufacturing practice facilities. The cord blood (CB) was obtained from mothers with vaginal or cesarean section delivery after informed consent. The parents sign a consent in which they are informed about the collection and storage procedures and which specifies the transplant and non-transplant uses of CBS. Informed consent is part of an Italian legislative decree, approved by the privacy guarantor, and is the same for all Italian banked samples.

The collection in the delivery room is carried out according to clinically validated procedures internal to the banked samples. Mothers’ peripheral blood was collected after birth in a vacutest tube without any anticoagulant, and the tests were immediately performed to detect the presence of infective diseases (hepatitis B and C virus, human immunodeficiency virus, TPHA, TOXO, CMV, HIV/HBV/HCV-NAT, and HTL VI/II). All the CB units had been collected after a donor selection questionnaire based on international criteria for cord blood banking. All steps from the recruitment to the processing and registration of CB for transplantation were performed according to standard operating procedures and guidelines edited by the Foundation for the Accreditation of hematopoietic cellular therapy (FACT). CB collection was achieved when the placenta was still in utero. Moreover, to prepare eye drops, blood samples were collected from ex-uterus placenta vessels with a sterile syringe and transferred to vacutest tubes without any anticoagulant validated for human blood component collection. For further processing, the units and related samples were sent to the processing facility laboratory. Blood was clotted for two hours at room temperature. After centrifugation at 3000 rpm for 15 min, the serum was carefully isolated under a laminar flow hood (BacT/Alert; Biomérieux, Marcy-l′Étoile, France; sterility tests were performed in each batch). We performed a screening of the CBS to be used as the eye drop preparation source for corneal disease treatment by EGF content analysis (Elisa Kit Quantikine; R&D Systems, Abingdon, UK) before CBS storage (EGF content > 1.5 ng/mL selected as the threshold). Sera were then frozen at −80 °C for the quarantine period. After the quarantine, the preselected sera were thawed and pooled to obtain the amount of serum needed to treat all animals and then filtered (Millex-HV Syringe Filter Unit, 0.45 μm pore size, Millipore, Burlington, MA, USA). The preparation was then aliquoted into a COL-20 medical device (Biomed, Modena, Italy) in single-dose vials, packed, frozen, and stored. CBS has been used under the ethical authorization: “Cord Blood Serum in the Treatment of Neuro-Degenerative Ophthalmic Diseases” Glaucoma CE code 128/2017/U/Sper; ClinicalTrials.gov Identifier: NCT03609125.

### 2.2. Growth Factor and Interleukin Dosage

We retained one aliquot for further testing of selected growth factors (GFs), in particular, IL-10, IL-13, basic FGF, PDGF-bb, b-NGF, EGF, and TGF-α. Samples were evaluated by using commercially available multiplex bead-based sandwich immunoassay kits (Bio-Rad Laboratories, Inc., Hercules, CA, USA) in the Bio-Plex Protein Array System (Bio-Rad Laboratories) as previously described [14]. This system consents a simultaneous quantitative analysis of multiple different factors in a single microliter well. Briefly, we used different sets of fluorescently dyed beads, loaded with capture monoclonal antibodies, specific for each tested cytokine. We measured and quantified the formation of different sandwich immune complexes on distinct bead sets. We discarded values with a coefficient of variation above 10% before performing data analysis with the Bio-Plex Manager software (version 6.0, Bio-Rad Laboratories). We considered only standard levels between 70% and 130% of the expected values. Eye drops used in the present experiments contained the following GFs (pg/mL), as reported in Table 1.

We treated the animals four times a day, administrating a drop of approximately 15 microliters per time, so meaning that each day, the treated eye received approximately: 

EGF: 53.4 pg; TGF-α: 3.996 pg; PDGF-bb: 426.18 pg; FGF-basic: 4.65 pg; bNGF: 0.042 pg; IL10: 0.924 pg; and IL13: 15.762 pg.

### 2.3. Animal Models and Experimental Procedure

All the experiments reported were performed following the ARVO Statement for the Use of Animals in Ophthalmic and Vision Research, under the protocol approved by the Ministry of Health (authorization number 862/2018-PR).

Albino rats (Sprague-Dawley) were born and raised under an ambient luminance level of approximately 5 lux (12 h light, 12 h dark), with food and water available ad libitum. The data here reported were obtained from experiments conducted on 20 animals aged between 2 and 4 months. To guarantee that all the animals used in this study were healthy and homogeneous, we recorded electroretinographic responses two weeks before starting the eye drops treatment. After this assortment, we divided the selected animals into four groups: 5 light damaged (LD control), 5 light damaged with eye-drop treatment in the right eye (LD RE treated) and the same quantitative of saline in the fellow eye (LD LE untreated), 5 healthy controls (HC) and 5 healthy animals treated with CBS eye drops in the right eye (HC treated).

### 2.4. Eye-Drop Treatment

The treatment was started seven days before light-damage, and it was dispensed up to the sacrifice of the animal. In order to minimize the variability between animals, 15 μL of CBS eye drops were supplemented using a Gilson pipette (Gilson Inc., Middleton, WI, USA) directly on the right eye (RE) cornea of the rats, four times/per day, after having thawed one vial the evening before. We used the left eye (LE) as an internal control, administering 15 μL of saline solution. After light damage, rats were returned to dim-circadian conditions and treated with the same protocol for a further seven days. At the end of this period, we recorded flash electroretinograms (f-ERG), and immediately after, we sacrificed the animals to collect tissues for the following analyses.

### 2.5. Light Damage

Albino rats were moved singularly into a cage with illumination from top and bottom to ensure the iso-luminance environment (1000 lux). We removed the litter from the cage to prevent rats from hiding from the light. Light damage started at the beginning of the day phase in the animal house, therefore, immediately after 12 h of darkness. During light damage, we exposed the animals to 1000 lux light for 24 h consecutively, and after we placed them back into standard cages and re-stabled in normal conditions.

### 2.6. Electrophysiological Recordings

To minimize the variability among experimental groups, we performed electrophysiological recordings (f-ERG) before exposing the animals to high-intensity light (LD). The visual function was re-evaluated one week after light damage in both treated (*n* = 5 RE treated and *n* = 5 LE untreated) and untreated LD animals (*n* = 5 LD control). Albino rats were dark-adapted for 12 h overnight, and electroretinograms were recorded in a completely darkened room [15]. Briefly, animals were anesthetized with Ketamine/Xylazine (Ketavet 100 mg/mL, Intervet production s.r.l., Aprilia-Latina, Italy; Xylazine hydrochloride X1126 Sigma-Aldrich Co., St. Louis, MO, USA at 10 mg/100 g–1.2 mg/100 g) intraperitoneal injection, fixed in a stereotaxic apparatus, and placed inside the Ganzfeld dome (Biomedica Mangoni, Pisa, Italy). We monitored and maintained the body temperature at 37.5 °C using a heating pad controlled by a rectal temperature probe. Corneas were anesthetized with a drop of Oxybuprocaine Chlorhydrate 4 Mg/mL (Novesine, Thea Farma Spa, Milano, Italy), and pupils were dilated with 1% atropine sulfate (Allergan, Westport, CO. Mayo, Ireland). This electronic flash unit generates flashes of intensities ranging from 0.001–100 cd/m^2^. Responses were recorded for 300 ms plus 25 ms of pretrial baseline, differentially amplified, bandpass filtered (0.3–300 Hz), and digitized at 0.25–0.3 ms intervals using a custom LabVIEW 8.2 routine (National Instruments, Milan, Italy). Responses recorded at increasing light intensities were averaged (*n* = 3), with an inter-stimulus interval (ISI) ranging from 60 s for dim lights to 5 min for the three brightest flashes. We analyzed: a-wave, b-wave, and oscillatory potentials. We used custom-written procedures in IGOR Pro 6.3 (WaveMetrics Inc., Lake Oswego, OR, USA) software to analyze the electrophysiological data.

### 2.7. Morphological Analyses and Immunostaining

After the last electroretinogram recording, we euthanized the rats and enucleated the eyes for morphological analyses. Eyes were dissected and fixed by immersion in 4% paraformaldehyde fixative buffer at 4 °C for 1 h, rinsed three times in 0.1 M phosphate-buffered saline (PBS) and cryoprotected by immersion in 10%, 20%, and left overnight in 30% sucrose solution. Eyes were then embedded in mounting medium (Tissue Tek^®^ OCT compound; Sakura Finetek, Torrance, CA, USA) by briefly freezing them in liquid nitrogen. We cut cryosections at 20 µm thickness (CM1850 Cryostat; Leica, Wetzlar, Germany) with the eyes oriented so that the sections extended from center to periphery. Sections mounted on gelatin and poly-L-lysine coated slides were then dried overnight in an oven at 50 °C and stored at −20 °C until processed.

All the sections were labeled with the nuclear dye bisbenzimide (Calbiochem, La Jolla, CA, USA) for 2 min (1:10.000 in 0.1 M PBS). We selected only the sections cut adjacent to or through the optic nerve head, to minimize variations in retinal length and position. Each section was scanned from the center to the peripheral edge of the retina.

We estimated photoreceptor survival by measuring the thickness of the outer nuclear layer (ONL). We measured the width of the ONL across the entire retinal extension from dorsal to ventral crossing the papilla, averaging four measurements performed every 0.40 mm interval, as shown in Figure 2. We used the ratio of ONL to the retinal thickness to measure ONL thickness, rather than the absolute width of the ONL (mm), to compensate for possible oblique sectioning. All measurements were performed using ImageJ 2.0 software.

We also immunolabeled retinal sections for the fibroblast growth factor (FGF2), ionized calcium-binding adaptor molecule 1 (IBA-1), and glial fibrillary acidic protein (GFAP) protein. We used 1% bovine serum albumin (BSA) and 10% goat serum for FGF2 and IBA-1, respectively, to block nonspecific binding. We incubated the sections overnight at 4 °C with mouse monoclonal anti-FGF-2/basic FGF antibody (EMD Millipore, Burlington, Massachusetts, USA, cat#05-117; diluted 1:200 in 0.75% HS), rabbit-polyclonal IBA1 (Wako Pure Chemical Industries, Ltd. Reagent Research Laboratories, Tokyo, Japan; diluted 1:1000 in 0.3% Triton X-100 in 1% goat serum), and polyclonal rabbit GFAP (Dako, Santa Clara, CA, USA, cod.n. Z0334; diluted 1:5000 in 0.75% horse serum). Secondary antibodies were anti-mouse, and anti-rabbit IgG conjugated to a green fluorescent dye (Alexa Fluor 488; Molecular Probes, Invitrogen, Carlsbad, CA, USA) diluted 1:200 and incubated at 37 °C for 2 h.

Quantification of FGF2 and GFAP fluorescence was performed by averaging the measurements obtained from 5 ROIs of 25 pixels × 25 pixels placed in different positions: throughout the entire ONL, for FGF2, and the whole retina for GFAP measurements. IBA-1-positive cells were counted throughout three complete retinal slices for each eye, only in the ONL. Microglial cells in a quiescent state look very arborized and become more amoeboid in a reactive state [16]. We performed Scholl analysis by using the free software Fiji default plugin. Briefly, we counted the number of intersections obtained between microglial arborization and concentric circles of expanding diameter (one every 5 pixels corresponding to 0.38 µm) drawn from the center of the analyzed microglia, sampled from the overall retina layers. We calculated the microglia area and the perimeter after manual thresholding of the image to separate the analyzed cell from the background.

### 2.8. Statistical Analysis

**Figure 1:**
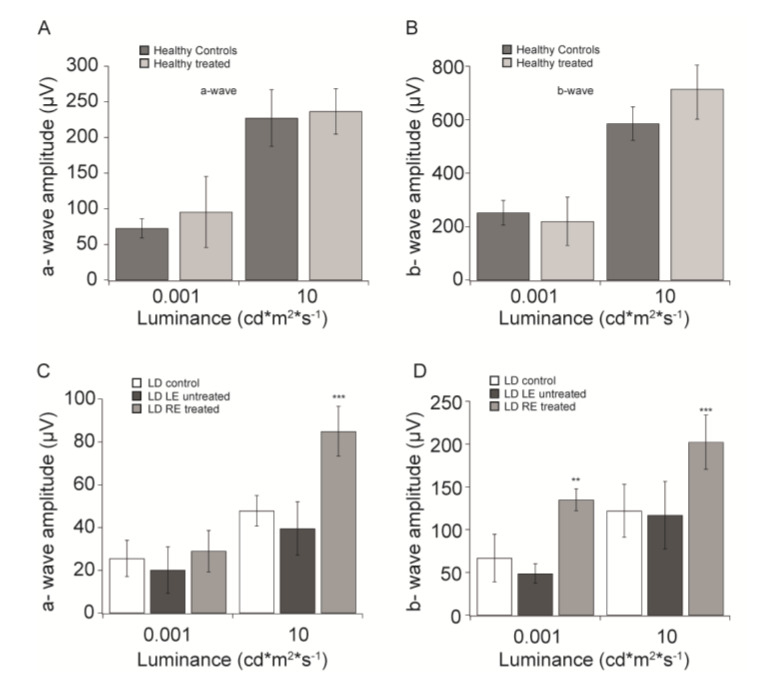
CBS treatment preserves retinal function. (**A**) a-wave amplitudes (mean ± SD) recorded from healthy controls (HC, black) and CBS treated healthy animal (HC treated, gray) in response to 0.001 and 10 cd*m2*s-1 flashes. (**B**) b-wave amplitudes (mean ± SD) recorded from healthy controls (HC, black), and CBS treated healthy animal (HC RE treated, gray) in response to 0.001 and 10 cd*m2*s^−1^ flashes. (**C**) a-wave amplitudes (mean ± SD) recorded from light damaged animals with no CBS treatment (LD control, white), the untreated eye of a light damaged animal (LD LE untreated, black) and CBS treated eye of a light damaged animal (LD RE treated, gray) in response to 0.001 and 10 cd*m2*s^−1^ flashes. (**D**) b-wave amplitudes (mean ± SD) recorded from light damaged animals with no CBS treatment (LD control, white), the untreated eye of a light damaged animal (LD LE untreated, black) and CBS treated eye of a light damaged animal (LD RE treated, gray) in response to 0.001 and 10 cd*m2*s^−1^ flashes. ** *p* < 0.01; *** *p* < 0.001.

**Figure 2:**
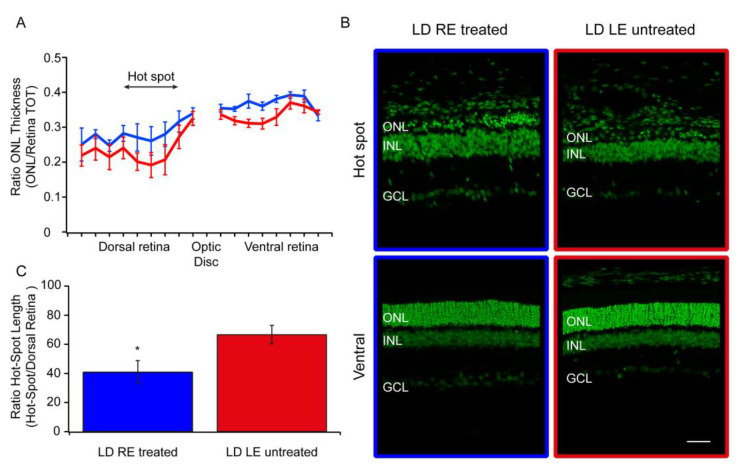
CBS treatment preserves retinal morphology. (**A**) The graph shows retinal outer nuclear layer (ONL) thickness measured in treated (LD RE treated, blue) and untreated (LD LE untreated, red) animals both in the dorsal and ventral region. ONL thickness is expressed as a ratio of ONL thickness to the retinal thickness to compensate for possible oblique sectioning. (**B**) Treated (LD RE treated, blue frame) and untreated (LD LE untreated, red frame) retinas stained with Hoechst to visualize the retinal nuclear structure in the hot-spot and ventral area, respectively. Scale bar: 50 µm. (**C**) Hot-spot lateral extension measured as the ratio of hot-spot length to dorsal retina length: treated (LD RE treated, blue) and untreated (LD LE untreated, red). * *p* < 0.05.

**Figure 3:**
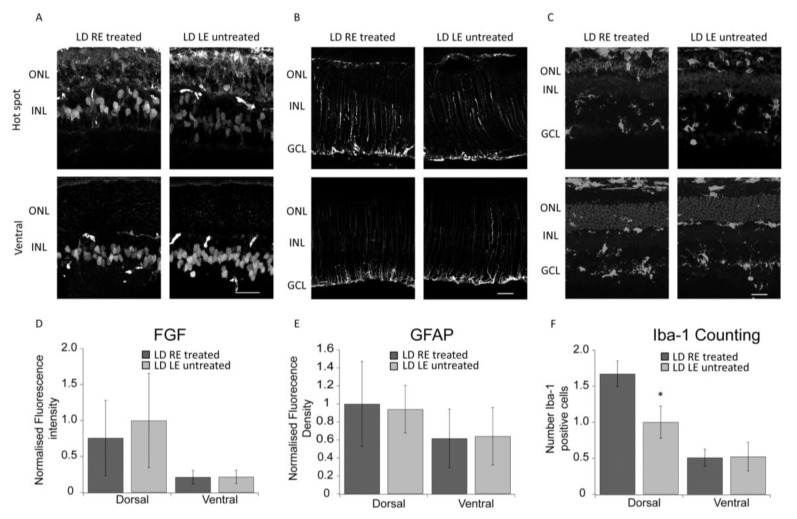
CBS treatment reduces microglia migration in the ONL. (**A**) Treated (LD RE treated) and untreated (LD LE untreated) retinas marked for FGF2. The upper panel shows the “hot spot” area in the dorsal retina, the bottom panel, a representative portion of the ventral retina. (**B**) Treated (LD RE treated) and untreated (LD LE untreated) retinas marked for GFAP. The upper panel shows the “hot spot” area in the dorsal retina, the bottom panel, a representative portion of the ventral retina. (**C**) Treated (LD RE treated, black) and untreated (LD LE untreated, gray) retinas marked for IBA-1. The upper panel shows the “hot spot” area in the dorsal retina, the bottom panel, a representative portion of the ventral retina. (**D**) FGF2 levels, measured in the ONL, in both treated (LD RE treated, black) and untreated (LD LE untreated, gray) retinas. (**E**) GFAP levels, measured along with the entire thickness of the retina, of treated (LD RE treated, black) and untreated (LD LE untreated, gray) retinas. (**F**) The number of microglial cells in the ONL of treated (LD RE treated, black) and untreated (LD LE untreated, gray) retinas (* *p* < 0.05). Scale bars in panel A, B, and C = 30 µm.

**Figure 4:**
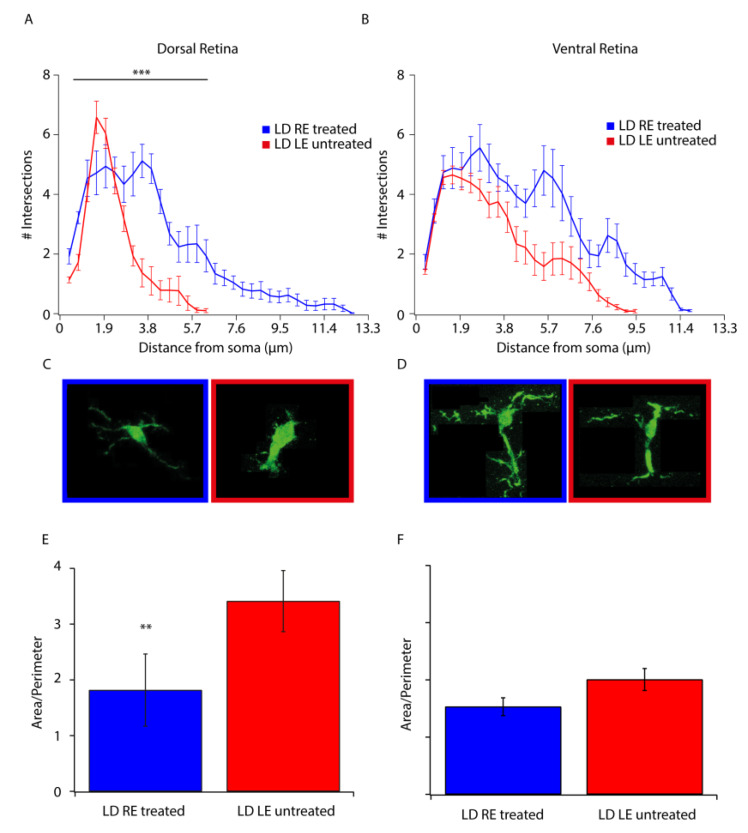
Microglial activation is reduced upon CBS treatment. (**A**,**B**) The number of intersections between microglial cell profile and concentric circles of expanding diameter (every 5 pixels corresponding to 0.38 µm) centered on the soma of the analyzed cell, in the dorsal (**A**) and ventral (**B**) retina of treated (LD RE treated, blue) and untreated (LD LE untreated, red) eyes after seven days from the light damage. (**C**,**D**) Representative microglial morphology in the dorsal (**C**) and ventral (**D**) retina in treated (LD RE treated, blue frame) and untreated (LD LE untreated, red frame) eyes. (**E**,**F**) Histograms showing the area to perimeter ratio calculated for microglial cells in the dorsal (**E**) and ventral (**F**) retina of treated (LD RE treated, blue) and Untreated (LD LE untreated, red) eyes. ** *p* < 0.01; *** *p* < 0.001.

The statistical analysis for all the data presented in Figure 1, Figure 2, Figure 3, and Figure 4E,F have been calculated using the two way ANOVA followed by the Tukey test.

Scholl analysis profile presented in Figure 4A has been analyzed by nonlinear regression fitting with least square regression a Gaussian curve and comparing or whether the best-fitting values of mean, SD, and amplitude are significantly different or if one curve could adequately fit all curves. Both calculated comparisons rejected the null hypothesis with *p* < 0.0001.

## 3. Results

### 3.1. “In vivo” Functional Analysis

To exclude undesired adverse reactions on healthy rats, we firstly compared the electroretinographic responses to flashes of increasing intensities (f-ERG) in the dark-adapted condition of healthy animals treated (HC treated) for seven days with CBS eye drops to those of untreated (HC) healthy animals.

Figure 1A,B shows the amplitudes of a- and b-waves recorded at two flash intensities (0.001 and 10 cd*m^2^*s^−1^), from healthy eyes with and without eye drops treatment. Both treated and untreated eyes show responses of comparable amplitude.

We then evaluated retinal function one week after exposure to damaging light in animals continuously treated from seven days before LD to seven days after LD. Figure 1C,D shows a- and b-wave amplitudes recorded for treated (LD RE treated), untreated fellow (LD LE untreated), and control LD eyes (LD control). In agreement with previously obtained results, light-damage induces a sharp reduction of both a- and b-waves amplitude in control animals. Interestingly, we did not observe a significant difference in the a-wave magnitude, at scotopic luminance (0.001 cd*m^2^*s^−1^), between treated and untreated eyes and LD control group (Figure 1C). Nevertheless, at the same luminance, in Figure 1D, CBS treatment significantly preserved the b-wave compared to both untreated (LD LE untreated) and control damaged animals (LD control; *p* < 0.01). At photopic luminance (10 cd*m^2^*s^−1^), instead, CBS-treated eyes show significant (*p* < 0.001) larger a-wave amplitude compared to LD control and untreated eyes (Figure 1C) and a similar trend is observable also for the b-wave in Figure 1D (*p* < 0.001).

We did not find significant effects in oscillatory potentials (data not shown).

### 3.2. Morphological Analysis

In LD retinas, photoreceptors initially die in the “hot spot” area, and neuronal death increases progressively in adjacent areas, resulting in a markedly reduced width of the outer nuclear layer (ONL). We measured the width of the ONL along the entire retina from dorsal to ventral crossing the optic disk reporting the data in Figure 2. In the dorsal retina of treated eyes (LD RE treated), the ONL is more preserved than the fellow eye (LD LE untreated), while the ventral retina appeared similar in the two groups (Figure 2A). Representative images were taken in corresponding “hot spot” retinal regions in both LD LE untreated and LD RE treated eyes (Figure 2B). Besides, we expressed the extension of the “hot spot” as a ratio between the length of the “hot spot” and the total dorsal length (Figure 2C). The extension of the damage was significantly reduced in treated eyes compared to fellow eyes.

### 3.3. Stress and Neuroinflammation Markers

The stress of any origin induces a retinal response, as immediately shown by Müller cells activation. Increased expression in GFAP is a sign of the reactive response of Müller cells, which orchestrate down-stream events by releasing several factors inducing activation of a self-protective mechanism (FGF2) as well as a neuroinflammatory reaction (microgliosis) [16]. Figure 3A–C shows images of GFAP, FGF2, and IBA-1 immunolabeled treated (LD RE treated) and untreated (LD LE untreated) retinas. No significant differences can be detected in GFAP and FGF2 images between treated and untreated retinas. While there was no apparent modulation in the stress response, migration of microglia (visualized trough Iba-1 staining) from the inner to outer retina was highly reduced. Indeed, in a healthy retina, microglia were resident in the inner side and moved to the outer in response to stress signals and to clear the ONL of dying cells. Figure 3C–E reports quantified measures of fluorescence and microglial cell numbers in the outer retina. In treated retinas (LD RE treated), migration of microglia appeared reduced in the dorsal retina, while no differences were observed in the ventral side. Morphometric studies in Figure 4 strengthened this point. Scholl analysis provides quantitative evidence of the activation of microglia. Our analysis revealed that microglial cells in CBS treated retinas were less reactive. Indeed the resting form of microglia is characterized by long branching processes and a small body distinguishable from the reactivate ones (more rounded) [17], characteristic of LD LE untreated retinas. Interestingly treatment seems to control microglia activation in the entire retina (dorsal and ventral).

## 4. Discussion

Present results seemed to confirm the hypothesis that cord blood serum (CBS) based eye drops might be successfully used to mitigate retinal neurodegenerative processes. The function was partially maintained, and retinal morphology looked in better shape compared with untreated fellow eyes and LD control eyes. Interestingly, the expected cross-talk [18,19] between the two eyes is minimal, if any, according to our data. We did not have a simple explanation for these results, but we could only hypothesize that eye drop components act directly on their target receptors with no spillover along optic nerve fibers or vascular system. The stress response of retinal tissue is not modulated, so there is no activation of self-protective mechanisms (see [17] for reference). Microglia migration to the outer nuclear layer (ONL) was significantly reduced as well as microglial activation, evidenced by the Sholl analysis. Microglia activation leads to neuroinflammation and the progression of neurodegenerative events. Recently a paper published from our group [13] reports a preliminary observation on two glaucomatous patients, which supports our hypothesis of successful therapy with CBS. The unifying pathophysiology characteristics between glaucoma and AMD are the activation of neuroinflammatory events, which are responsible for the progression of both diseases. Our data provided evidence of maintenance of function together with a reduced microglia activation/migration, primary events in neuroinflammation progression. Two trophic factors have been shown to cope successfully with neurodegeneration: NGF in animal models and patients [9,20,21,22] and BDNF in animals [10]. Here we show that a natural combination of cytokines and trophic factors can ameliorate retinal function and morphology in the stressed retina. It has to be noted that these trophic factors are present in CBS with a concentration ten times lower than those tested in both animal models and patients. Altogether these data support the possibility to transfer CBS eye drops treatment to patients to cope with and mitigate neurodegenerative retinal diseases.

## 5. Conclusions

In our experiments, only a limited number of factors in CBS were determined, which used the original protocol that was developed to be used in corneal epithelial defect treatment. Nevertheless, it has to be noted that CBS with similar tested contents gave promising preliminary results in glaucomatous patients [13]. It remains an open question whether some factors might contribute more efficiently to achieve neuroprotection. Accordingly, we are increasing the panel of cytokines and grow factors screened in the new preparations for the ongoing experiments. Indeed, in future experiments, our target will be to correlate CBS content with treatment efficacy. The combination of different trophic factors at different concentrations might allow us to better define the right treatment for selected pathologies.

## 6. Patents

A patent covering the topic of this manuscript has been filed (WO2018229718) on December 20, 2018, and is owned by the University of Bologna, AOU di Bologna S. Orsola Malpighi Teaching Hospital, and the University of L’Aquila.

## Figures and Tables

**Table 1:** Quantification of selected growth factors in CBS.

EGF	TGF-α	PDGFbb	FGF Basic	b-NGF	IL-10	IL-13
890	66.6	7103	77.5	0.70	15.4	262.7

^1^ Quantification of selected growth factors in the used cord blood serum (CBS) eye drops expressed in picograms/milliliter.

## List of Abbreviations:

AMD	Age-Related Macular degeneration
BDNF	Brain-derived neurotrophic factor
CB	cord blood
CBS	cord blood serum
CMV	Cytomegalovirus
EGF	Epidermal growth factor
fERG	flash Electroretinogram
FGF basic	Fibroblast growth factor
GCL	ganglion cell layer
GF	Growth factor
GFAP	Glial fibrillary acidic protein
HIV/HBV/HCV-NAT	Human Immunodeficiency Virus/Hepatitis B virus/Hepatitis C Virus -Nucleic Acid Testing
HTL	VI/II human T-lymphotropic virus I/II
IBA-1	Ionized calcium-binding adaptor molecule 1
IL-10 and -13	Interleukin-10 and -13
INL	inner nuclear layer
LD	Light damage
LE	left eye
NGF	Nerve growth factor
OCT	optimum cutting temperature
ONL	outer nuclear layer
Ops	oscillatory potentials
PDGF-bb	Platelet-derived growth factor-bb
PBS	phosphate-buffered saline
RE	right eye
RPE	retinal pigmented epithelium
TGF-α	Transforming growth factor-α
TOXO	Toxoplasmosis
TPHA	Treponema pallidum hemagglutination (TPHA)
